# Current State of Clinical Trials for Keloid Management: An Analysis of Trials Registered on ClinicalTrials.gov

**DOI:** 10.7759/cureus.106243

**Published:** 2026-03-31

**Authors:** Sookyung Park, Deng Li, Linfen Guo, Xuewen Xu

**Affiliations:** 1 Department of Plastic and Burns Surgery, West China Hospital, Sichuan University, Chengdu, CHN

**Keywords:** clinical trials, clinicaltrials.gov, interventional study, keloid, registry analysis, results reporting

## Abstract

Background

Keloid management remains clinically challenging, and the clinical trial landscape underpinning keloid therapeutics has not been comprehensively characterized.

Objective

The objective of this study is to characterize the structural features of interventional keloid trials registered on ClinicalTrials.gov, including phase distribution, enrollment size, intervention category, sponsor type, and geographic representation.

Methods

We conducted a cross-sectional registry analysis of interventional keloid trials listed on ClinicalTrials.gov. Trials with study start dates between January 1, 2005, and December 31, 2024, were identified. Study phase, enrollment size, intervention category, sponsor type, geographic region, trial status, and registry results availability were extracted and descriptively analyzed. For completed trials, PubMed was searched using each trial’s National Clinical Trial (NCT) identifier to assess dissemination outside the registry.

Results

A total of 84 interventional keloid trials met the inclusion criteria. Among the 56 phase-classified trials, early-phase studies (Early Phase 1 through Phase 2) accounted for 62.5%, whereas late-phase studies (Phase 2/3 through Phase 4) accounted for 37.5%. Across all included trials, only six studies (7.1%) were Phase 3, and registry-reported enrollment was below 50 participants in 65 trials (77.4%). Drug-based interventions were the most common category (46.4%), whereas biologic and radiation modalities were rarely represented. Within ClinicalTrials.gov-registered trials, study locations were concentrated in North America and Europe. Regarding temporal distribution, 56 trials (66.7%) were initiated between 2015 and 2024. Of the 84 trials, 52 (61.9%) were classified as completed. Among these, only 10 (19.2%) had results posted on ClinicalTrials.gov, and PubMed searching identified three keloid-specific final results publications, yielding results available through either source for 13 completed trials (25.0%).

Conclusion

As reflected in ClinicalTrials.gov-registered trials, the current interventional keloid trial landscape is characterized by small studies, early-stage predominance, uneven modality representation, and limited geographic diversity. Limited results accessibility among completed trials further underscores structural gaps in evidence dissemination. These findings highlight the need for larger, more geographically inclusive trials with clearer outcome assessment frameworks and more consistent reporting of results.

## Introduction

Keloids are fibroproliferative scars characterized by chronic inflammation and excessive extracellular matrix deposition that extend beyond the boundaries of the original wound [1,2]. They can cause pain, pruritus, functional limitation, and cosmetic disfigurement, substantially impairing quality of life [1]. Keloids also occur disproportionately in certain populations, with the highest prevalence reported in people of color and estimated incidence ranging from 4% to 16% in populations of African ancestry [1,3].

Multiple therapeutic approaches have been proposed for keloid management, including intralesional corticosteroids, cryotherapy, surgical excision, radiation, laser therapy, and biologic or other emerging strategies [3,4]. However, no single modality has demonstrated consistently superior outcomes across clinical settings, and recurrence remains a major challenge [3]. Recent reviews have also highlighted heterogeneity in treatment protocols, outcome assessment measures, and follow-up duration, which complicates cross-study comparison and limits the development of stronger evidence-based recommendations [3,5,6]. Accordingly, persistent uncertainty in keloid management may reflect not only biological complexity but also limitations in how interventional evidence is generated and evaluated.

While prior reviews have summarized treatment outcomes and assessment methods [3, 5], less attention has been directed toward the structure of the interventional research landscape itself. In areas where therapeutic consensus remains limited, trial characteristics such as phase distribution, enrollment scale, intervention pattern, geographic representation, and sponsor type may influence the robustness and generalizability of the available evidence. Broader evaluations of trial informativeness have also suggested that design, feasibility, and reporting features are important determinants of how well clinical trials inform practice [7]. We therefore conducted a cross-sectional analysis of interventional keloid trials registered on ClinicalTrials.gov to characterize the structural features of the current clinical trial landscape. Specifically, this study asked whether patterns in phase distribution, enrollment scale, intervention profile, sponsorship, and geographic representation might help explain current limitations in the evidence base for keloids.

## Materials and methods

ClinicalTrials.gov was searched on December 10, 2025, using the ClinicalTrials.gov search interface. The query was restricted to the Condition/Disease field, in which “keloid OR Keloids” was entered. The study type filter was set to interventional studies, and the study start date range was limited from January 1, 2005, to December 31, 2024. ClinicalTrials.gov was selected as the primary registry because it is a large publicly accessible database of clinical studies and provides a standardized framework for identifying and characterizing interventional research [8].

This search yielded 99 records. Search results were downloaded in CSV format, including National Clinical Trial (NCT) number, study title, study status, study results, conditions, interventions, sponsor, phase, enrollment, study type, start date, completion date, first posted date, and locations. Observational studies and expanded access programs were excluded.

Records were then manually screened using the registry's Conditions field to retain interventional trials focused on keloids. Trials were retained if the Conditions field explicitly listed keloid or keloids, including records in which keloid appeared alongside other scar-related terms. Records were excluded when the Conditions field instead referred to non-keloid entities or broader scar-related conditions, including acne keloidalis nuchae, burn scar, cesarean section scar or wound, dermatologic disease, fibrosis, hypertrophic scar(s), skin scarring, tattoo-related scarring, or scar prevention/scar reduction. Trials investigating acne keloidalis nuchae were also excluded because this entity is clinically and pathophysiologically distinct from keloid disease [9]. After condition-field screening, 15 records were excluded, and 84 interventional keloid trials were included in the final analysis. For transparency and reproducibility, the excluded records and their registry-reported conditions entries are provided in Appendix A.

Data extraction was performed by one reviewer. Two additional reviewers independently checked trial eligibility and verified extracted data against the original ClinicalTrials.gov records. Any discrepancies or ambiguous coding decisions regarding eligibility, multi-country coding, duplicate handling, grouping decisions, or rounding were resolved by rechecking the original registry records and reaching consensus through discussion. A senior reviewer performed a final verification of the dataset. The prespecified analytic outcomes were registry-derived trial characteristics, specifically study phase, enrollment size, intervention category, sponsor type, geographic representation, trial status, and results availability.

Trials were characterized by study phase (Early Phase 1, Phase 1, Phase 1/2, Phase 2, Phase 2/3, Phase 3, Phase 4, or Not Applicable), enrollment size (0-9, 10-49, 50-99, 100-499, or ≥500 participants), intervention category, geographic region, and sponsor type (industry vs. non-industry). Intervention types were classified into seven mutually exclusive categories: (1) Drug, including systemic or topical pharmacologic agents; (2) Device, including energy-based systems, mechanical devices, or scaffolds; (3) Procedure, including surgical excision, cryotherapy, or other operative techniques; (4) Biologic, consisting of cell- or tissue-based therapies; (5) Radiation as a standalone modality; (6) Combination, defined as interventions incorporating two or more distinct modalities; and (7) Other, including non-therapeutic comparators such as moisturizers or educational interventions. Enrollment values were extracted as reported in ClinicalTrials.gov and may reflect either estimated or actual enrollment, depending on the trial record and update status.

Geographic reporting was performed at the trial-country pair level rather than the unique-country level. One country entry was counted per country listed in the registry for each trial. For multi-country trials, the country field was split by commas, each resulting entry was trimmed, and each entry was counted as one trial-country pair. Country-level entries were then mapped to four regions (North America, Europe, East Asia, and other regions). Because two trials were conducted in multiple countries, the number of geographic entries exceeded the number of trials: one trial contributed three country entries (Poland, the Bahamas, and the United Kingdom) and another contributed two (the United States and the Dominican Republic), resulting in 87 trial-country pairs across 84 trials.

For descriptive comparison of temporal patterns, trials were grouped into two time periods (2005-2014 and 2015-2024). Trial status was extracted from the ClinicalTrials.gov “Overall Status” field and categorized as completed, ongoing (recruiting, active not recruiting, enrolling by invitation, or not yet recruiting), terminated, withdrawn, or unknown. Results availability was assessed using the ClinicalTrials.gov “Has Results” field. For trials classified as completed, registry results availability was defined as the presence of posted results on ClinicalTrials.gov. For completed trials, publication outside the registry was additionally assessed by searching PubMed using each trial’s NCT identifier. PubMed was searched on March 11, 2026, in two passes for each completed trial: the NCT number as a standalone search term and the NCT number combined with the term “keloid.” Retrieved articles were considered matched publications only when they could be linked to the registered study by the NCT number and concordant study characteristics. Protocol papers, laboratory studies, and unrelated publications were not counted as final results publications.

## Results

After condition-field screening, 84 interventional keloid trials were included in the final analysis. A complete list of included interventional keloid trials is provided in Appendix B. The characteristics of included trials are summarized in Table 1.

**Table 1:** Characteristics of included interventional keloid trials Geographic region is reported as number (%) of trial-country pairs rather than unique countries; total trial-country pairs = 87 across 84 trials because two trials were conducted in multiple countries. All other characteristics are reported per trial (n = 84). Trials included in the analysis had study start dates between January 1, 2005, and December 31, 2024.

Characteristic	n (%)
Study Phase	
Early Phase 1	4 (4.8%)
Phase 1	5 (6.0%)
Phase 1/2	9 (10.7%)
Phase 2	17 (20.2%)
Phase 2/3	1 (1.2%)
Phase 3	6 (7.1%)
Phase 4	14 (16.7%)
Not Applicable	28 (33.3%)
Enrollment Size	
0–9	13 (15.5%)
10–49	52 (61.9%)
50–99	16 (19.0%)
100–499	3 (3.6%)
≥500	0 (0%)
Intervention Category	
Drug	39 (46.4%)
Device	18 (21.4%)
Combination	13 (15.5%)
Other	6 (7.1%)
Procedure	4 (4.8%)
Biologic	3 (3.6%)
Radiation	1 (1.2%)
Geographic Region	
North America	55 (63.2%)
Europe	12 (13.8%)
East Asia	5 (5.7%)
Other	15 (17.2%)
Sponsor Type	
Non-industry	64 (76.2%)
Industry	20 (23.8%)

Study phase distribution

**Figure 1:**
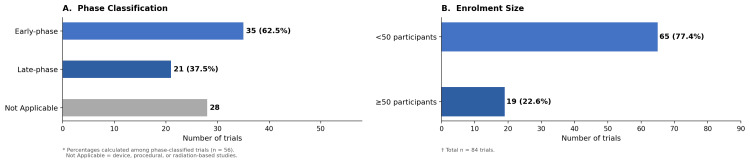
Structural characteristics of interventional keloid trials with study start dates between 2005 and 2024 (A) Phase classification of trials grouped into early-phase (Early Phase 1 through Phase 2) and late-phase (Phase 2/3 through Phase 4) categories. Percentages were calculated among phase-classified trials (n = 56). Not Applicable indicates studies that were not assigned a conventional phase designation in ClinicalTrials.gov. (B) Distribution of trials by registry-reported enrollment category (<50 vs. ≥50 participants).

Phase 2 studies constituted the largest proportion of trials (20.2%, n = 17), followed by Phase 4 (16.7%, n = 14) and Phase 1/2 (10.7%, n = 9) (Figure 1A). Early Phase 1 and Phase 1 trials represented 4.8% (n = 4) and 6.0% (n = 5), respectively, whereas Phase 2/3 studies accounted for 1.2% (n = 1) and Phase 3 studies for 7.1% (n = 6). Among the 56 phase-classified trials, early-phase studies predominated, accounting for 62.5% of classified trials. A substantial fraction of all included trials (33.3%, n = 28) were categorized as “Not Applicable,” indicating that they were not assigned a conventional phase designation in the registry.

Enrollment size

The majority of studies (61.9%, n = 52) had registry-reported enrollment values of 10-49 participants, whereas 15.5% (n = 13) had values below 10 participants. Moderate-sized studies (50-99 participants) accounted for 19.0% (n = 16), and only 3.6% (n = 3) had registry-reported enrollment values of 100-499 participants. No trial had a registry-reported enrollment value of 500 or more participants. Overall, registry-reported enrollment was below 50 participants in 65 trials (77.4%) (Figure 1B). Among the 52 completed trials, 49 trials were labeled as actual enrollment, whereas three remained listed as estimated in ClinicalTrials.gov.

Intervention categories

Drug-based interventions were the most common category, accounting for 46.4% (n = 39) of all included trials. Device-based therapies accounted for 21.4% (n = 18), and combination interventions incorporating two or more modalities accounted for 15.5% (n = 13). Other interventions represented 7.1% (n = 6), followed by procedural interventions at 4.8% (n = 4), biologics at 3.6% (n = 3), and radiation at 1.2% (n = 1). Overall, drug-based interventions predominated, whereas biologic and radiation-based approaches remained rare.

Geographic distribution

These 84 trials corresponded to 87 trial-country pairs after splitting multi-country trials into individual country-level entries. The majority of trial-country pairs were in North America (55/87, 63.2%), followed by Europe (12/87, 13.8%), East Asia (5/87, 5.7%), and other regions (15/87, 17.2%).

Sponsorship

Non-industry sponsors, including academic institutions, hospitals, and government agencies, accounted for 76.2% (n = 64) of trials, whereas industry sponsors represented 23.8% (n = 20).

Temporal distribution of trial initiation

**Figure 2:**
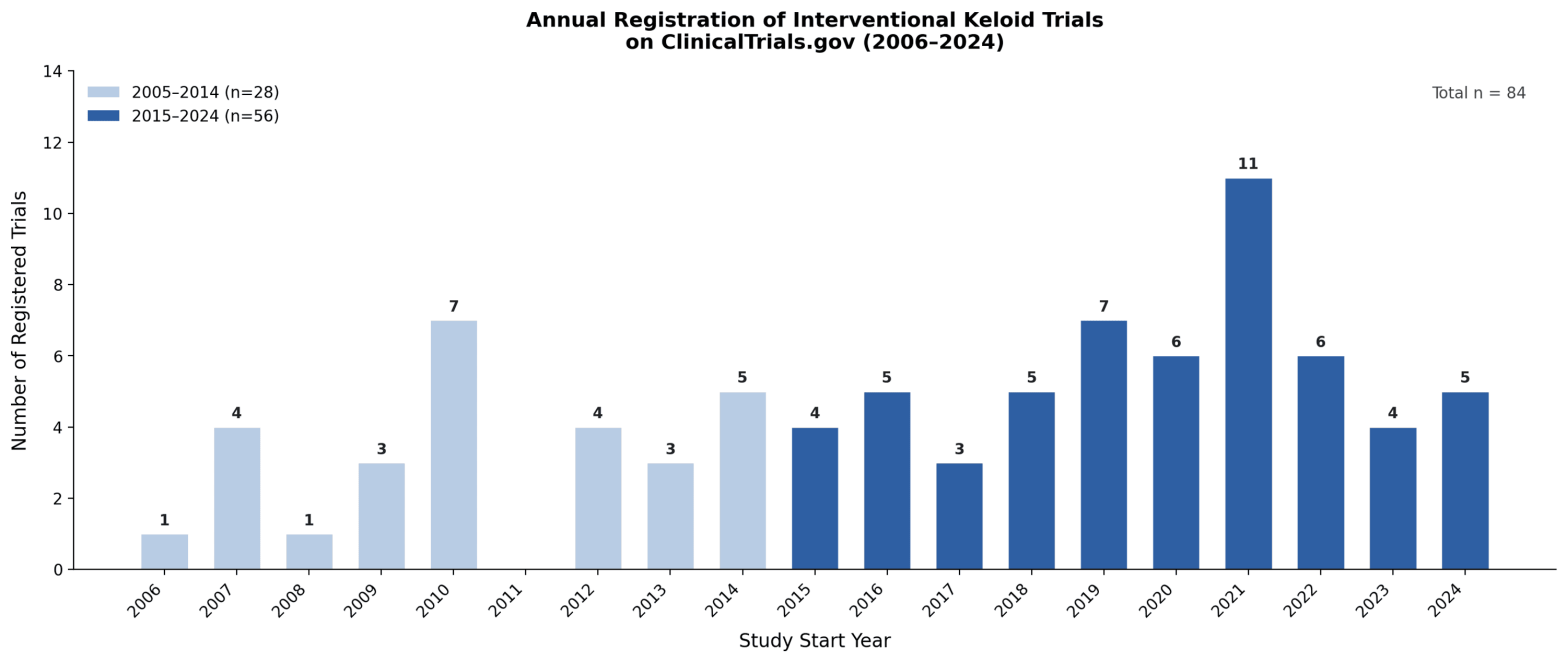
Annual registration of interventional keloid trials on ClinicalTrials.gov by study start year Bars represent the number of interventional keloid trials registered on ClinicalTrials.gov by study start year between 2006 and 2024. Bars are shaded to distinguish trials initiated during the 2005–2014 and 2015–2024 study periods. No included trial was initiated in 2005.

Trial initiation activity was concentrated in the most recent decade. Among all 84 included trials, 28 (33.3%) were initiated between 2005 and 2014, compared with 56 (66.7%) initiated between 2015 and 2024. The highest number of trial initiations in a single year was observed in 2021 (n = 11), and no included trials were initiated in 2005 or 2011 (Figure 2).

Trial status and results accessibility

**Figure 3:**
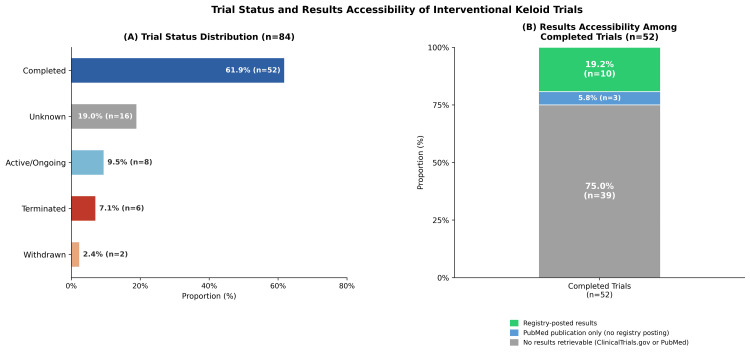
Trial status and results accessibility of interventional keloid trials (A) Distribution of trial status among all included trials (n = 84). Active/Ongoing includes recruiting, active not recruiting, enrolling by invitation, and not yet recruiting trials. (B) Results accessibility among completed trials (n = 52). Ten completed trials (19.2%) had results posted on ClinicalTrials.gov, three additional trials (5.8%) had confirmed keloid-specific final results publications identified through PubMed searching despite no posted registry results, and 39 trials (75.0%) had no results available through either ClinicalTrials.gov or PubMed.

Of the 84 included trials, 52 (61.9%) were classified as completed, 16 (19.0%) had unknown status, eight (9.5%) were ongoing, six (7.1%) were terminated, and two (2.4%) were withdrawn (Figure 3A). Among the 52 completed trials, 10 (19.2%) had results posted on ClinicalTrials.gov. To assess dissemination outside the registry, PubMed was additionally searched for all completed trials using each trial’s NCT identifier. Among the 42 completed trials without posted registry results, this search identified seven unique PubMed records. Three were confirmed as keloid-specific final results publications, whereas the other four consisted of one broader scar-related/device study excluding spontaneous keloids, two protocol-only publications, and one in vitro report without final clinical trial results. Across all 52 completed trials, 10 had results posted on ClinicalTrials.gov, and an additional three had confirmed final results publications identified through PubMed despite lacking posted registry results. Thus, results were available through either ClinicalTrials.gov or PubMed for 13 trials (25.0%), whereas 39 trials (75.0%) had no results available through either source (Figure 3B).

## Discussion

In this cross-sectional analysis of interventional studies registered on ClinicalTrials.gov, the contemporary keloid trial landscape revealed several recurring structural patterns. Most included studies had registry-reported enrollment below 50 participants, and study locations showed limited geographic diversity. Among phase-classified studies, early-phase trials predominated, whereas late-phase development remained comparatively uncommon. In addition, registry-posted results were available for only a minority of completed trials, and PubMed searching identified only three keloid-specific final result publications. Taken together, these features suggest that the current keloid evidence base is shaped less by mature confirmatory research than by fragmented, early-stage, and unevenly distributed investigation, which may help explain why therapeutic consensus in keloid management has remained difficult to establish despite the wide range of available treatment approaches.

The phase distribution and enrollment profile observed in this study provide one possible explanation for persistent uncertainty in keloid management. Among the 56 phase-classified trials, early-phase studies predominated. Across all included trials, only six studies (7.1%) were Phase 3, indicating relatively limited progression to confirmatory evaluation. Broader analyses of registered clinical trials have documented substantial proportions of small studies and evolving phase structures across the ClinicalTrials.gov ecosystem [10]. These observations are also consistent with keloid-specific reviews that have long emphasized limitations in the quality and consistency of the available therapeutic literature, as well as the continued need for larger and more robust studies to strengthen treatment recommendations [3,11]. At the same time, more than three-quarters of included trials fell below 50 participants, a pattern that may reduce statistical power, widen uncertainty around treatment effects, and complicate comparison across interventions. Smaller trials have also been shown to yield larger treatment effect estimates than larger studies in meta-epidemiological analyses [12]. Although late-phase development was not entirely absent, identified late-phase studies such as NCT02823236 suggest that confirmatory-stage investigation has occurred only selectively within the broader field [13]. However, this interpretation should be viewed in light of the study’s descriptive design and the absence of a formal assessment of trial quality.

The intervention profile observed in this study also suggests an uneven distribution of therapeutic development across modalities. Drug-based interventions accounted for the largest proportion of registered studies, whereas biologic and radiation-based approaches were rarely represented. This pattern may partly reflect the longstanding clinical reliance on pharmacologic approaches such as intralesional corticosteroids and related adjunctive regimens in keloid management [3,4]. At the same time, substantial heterogeneity in treatment protocols, outcome assessment methods, and follow-up practices continues to limit cross-study comparison. Keloid-focused reviews have identified numerous assessment measures, with only a limited number specifically designed for keloids [5], whereas broader scar-assessment reviews have similarly highlighted the lack of a universally accepted measurement framework across scar studies [14]. The low representation of biologic strategies in the registry is notable because recent therapeutic reviews have increasingly emphasized emerging targeted and biologically oriented approaches in keloid management [15]. However, when such trials were identified, they were often small in scale, as illustrated by the Phase 2 study of intralesional bevacizumab in keloid tissue (ClinicalTrials.gov identifier: NCT01408953) [15,16].

Geographic distribution and sponsor structure represent additional features of the current keloid trial landscape that may influence the breadth and applicability of the available evidence. In our ClinicalTrials.gov-based dataset, study locations were concentrated primarily in North America and Europe, although this pattern should be interpreted in light of the study’s reliance on a single registry. Broader evaluations of registered clinical trials have similarly shown that research activity is often concentrated in higher-income settings and may not align with health needs in non-high-income regions [17]. In the context of keloids, this mismatch may have practical implications beyond simple geographic imbalance. Because keloids disproportionately affect certain populations, underrepresentation of these groups in interventional research may limit the generalizability of the resulting evidence to the patients most affected by the disease [1,17]. Together with the predominance of non-industry sponsorship, these findings suggest that future progress in keloid research will depend on generating evidence from broader geographic settings and more representative patient populations.

The low rate of registry-posted results identified in this analysis represents an additional structural limitation of the current keloid trial landscape. Among the 52 completed trials, only 10 (19.2%) had results posted on ClinicalTrials.gov, indicating limited accessibility through registry-based reporting alone. Additional PubMed searching identified three keloid-specific final results publications, raising the proportion of completed trials with results accessible through at least one source to 25.0% (13/52); nonetheless, 75.0% of completed trials had no results available through either ClinicalTrials.gov or PubMed. This pattern is consistent with broader observations that results posted on ClinicalTrials.gov are frequently incomplete or delayed [18]. Taken together with the predominance of small, early-phase studies, these findings suggest that improving the completeness and accessibility of keloid trial reporting may be as important as increasing the volume of registered research.

This analysis was restricted to ClinicalTrials.gov and did not capture trials registered on other platforms such as the World Health Organization International Clinical Trials Registry Platform (WHO ICTRP), European Union (EU) Clinical Trials Register, or regional registries. As a result, the apparent geographic concentration in North America may be overestimated. Registry data also depend on the accuracy of sponsor-entered information, and fields such as study phase may be reported inconsistently. Because trial identification relied on explicit keloid indexing in the ClinicalTrials.gov condition fields, potentially relevant interventional records may have been missed if they were categorized only under broader scar-related terminology. Enrollment data were derived directly from ClinicalTrials.gov without systematic differentiation between estimated and actual figures. Some trial records may not have been updated after completion, so registry-reported enrollment may not always reflect final accrual, which could introduce imprecision into sample-size-based interpretations. The cross-sectional design limits causal interpretation of temporal changes and does not assess trial quality or comparative treatment efficacy. This study did not evaluate design features such as randomization, blinding, or outcome assessment, which are also relevant to the reliability and clinical applicability of the available evidence. Our additional publication search was limited to PubMed and relied primarily on NCT identifier-based matching. Therefore, some disseminated results may still have been missed if publications were not indexed in PubMed or did not report the trial registration number. In addition, results dissemination outside the registry was assessed through an additional PubMed search rather than as part of the primary registry-based analysis. Because many recently initiated trials remained ongoing or had unknown status at the time of data extraction, the present findings should be interpreted as a contemporary but still evolving snapshot of the registered keloid trial landscape.

## Conclusions

As reflected in ClinicalTrials.gov-registered trials, the current interventional keloid trial landscape is characterized not only by small studies, early-stage development, uneven modality representation, and limited geographic diversity but also by limited results accessibility among completed trials. Together, these features suggest that the apparent volume of registered keloid research may overstate the amount of evidence that is mature, accessible, and practically informative for clinical decision-making. Strengthening future evidence generation in keloid management will likely require not only larger and more geographically inclusive trials but also more complete and consistent reporting of trial results.

## Appendices

Appendix A 

**Table 2:** Excluded records after condition-field screening (n = 15) Conditions are reproduced from the registry's Conditions field as recorded in ClinicalTrials.gov at the time of data extraction.

NCT Number	Conditions
NCT06770946	Cesarean Section
NCT06729840	Scar, Hypertrophic
NCT06301178	Dermatologic Disease
NCT05608499	Acne Keloidalis Nuchae|AKN
NCT05259137	Hypertrophic Scar
NCT03306628	Skin Scarring
NCT02372786	Tattoo|Acne Keloidalis Nuchae
NCT01978301	Fibrosis
NCT01548898	Acne Keloidalis Nuchae
NCT01328080	Acne Keloidalis Nuchae
NCT01075165	Burn Scar
NCT00892723	Scar Prevention|Scar Reduction
NCT00849004	Hypertrophic Scars
NCT00825916	Scar Prevention|Scar Reduction
NCT00757315	Acne Keloidalis Nuchae|NdYag Laser|AKN|Acne Keloidalis|AK|Dermatitis Papillaris Capillitii|Folliculitis Keloidalis Nuchae|Sycosis Nuchae|Acne Keloid|Keloidal Folliculitis|Lichen Keloidalis Nuchae|Folliculitis Nuchae Scleroticans|Sycosis Framboesiformis

Appendix B 

**Table 3:** Summary of included interventional keloid trials registered on ClinicalTrials.gov, 2005–2024 NCT Number = ClinicalTrials.gov identifier; Country = country listed in the registry; Phase = registry-reported study phase; N = enrollment value as reported in ClinicalTrials.gov (estimated or actual, depending on the registry record); Interv. = intervention type as listed in the registry entry; Status = registry-reported study status at the time of data extraction; Results = whether results were posted on ClinicalTrials.gov at the time of data extraction; N/A = not applicable. Interv. is registry-reported and may differ from the harmonized categories used in Table 1.

#	NCT Number	Country	Phase	N	Interv.	Status	Results
1	NCT00469235	United States	PHASE1|PHASE2	20	Drug	Completed	No
2	NCT00519493	United States	N/A	3	Procedure	Terminated	No
3	NCT00565552	Germany	N/A	20	Drug	Unknown	No
4	NCT00587587	United States	PHASE1|PHASE2	30	Device	Completed	Yes
5	NCT00710333	United States	PHASE1|PHASE2	10	Drug	Completed	No
6	NCT00754247	United States	PHASE4	30	Drug	Completed	Yes
7	NCT00836147	United States	PHASE1|PHASE2	20	Drug	Completed	No
8	NCT00987545	United States	PHASE2	3	Drug	Terminated	No
9	NCT00993005	Cuba	PHASE3	90	Other	Completed	No
10	NCT01078428	Thailand	PHASE4	38	Other	Completed	No
11	NCT01113125	Denmark	PHASE3	60	Drug	Unknown	No
12	NCT01125137	United States	N/A	20	Procedure	Completed	No
13	NCT01158196	France	PHASE3	60	Device	Unknown	No
14	NCT01176877	United States	N/A	40	Other	Completed	Yes
15	NCT01408953	United States	PHASE2	2	Biologic	Terminated	No
16	NCT01425216	United States	PHASE2	60	Drug	Terminated	No
17	NCT01446770	Bahamas	PHASE2	19	Device	Completed	No
18	NCT01706861	Poland, Bahamas, United Kingdom	N/A	51	Device	Completed	No
19	NCT01720056	Australia	PHASE4	14	Drug	Terminated	No
20	NCT01736969	United States	N/A	40	Device	Unknown	No
21	NCT01789346	United States	N/A	20	Device	Completed	Yes
22	NCT01861119	South Korea	PHASE3	90	Drug	Completed	No
23	NCT01941914	Denmark	PHASE1	7	Drug	Completed	No
24	NCT02063243	Netherlands	N/A	33	Procedure	Completed	No
25	NCT02079168	United States, Dominican Republic	PHASE2	16	Drug	Completed	No
26	NCT02340325	Canada	PHASE1	20	Drug	Completed	No
27	NCT02521402	United States	PHASE4	10	Biologic	Unknown	No
28	NCT02546076	Italy	PHASE2	0	Device	Withdrawn	No
29	NCT02630303	United States	PHASE1	60	Device	Completed	No
30	NCT02823236	Mexico	PHASE3	102	Drug	Unknown	No
31	NCT02922972	France	N/A	15	Device	Completed	No
32	NCT02962518	Germany	N/A	18	Combination	Completed	No
33	NCT02996097	United States	N/A	30	Combination	Completed	Yes
34	NCT03160053	Hungary	N/A	2	Device	Completed	No
35	NCT03228693	United States	N/A	48	Procedure	Completed	No
36	NCT03239964	Egypt	PHASE4	146	Combination	Completed	No
37	NCT03312166	France	N/A	31	Device	Completed	No
38	NCT03433222	United States	PHASE1	60	Device	Unknown	No
39	NCT03460431	China	N/A	30	Device	Completed	No
40	NCT03601052	United States	PHASE2	14	Drug	Completed	Yes
41	NCT03630198	United States	PHASE4	31	Drug	Completed	Yes
42	NCT03760250	United States	PHASE2	6	Drug	Terminated	Yes
43	NCT03795116	United States	PHASE2	30	Device	Completed	Yes
44	NCT03876548	United States	EARLY_PHASE1	0	Device	Withdrawn	No
45	NCT03887208	Poland	PHASE1|PHASE2	100	Combination	Completed	No
46	NCT03982862	Taiwan	PHASE4	20	Drug	Unknown	No
47	NCT04016610	United States	N/A	9	Device	Completed	No
48	NCT04049552	United States	EARLY_PHASE1	4	Drug	Completed	Yes
49	NCT04169438	United States	N/A	10	Other	Completed	No
50	NCT04169490	United States	N/A	75	Other	Completed	No
51	NCT04186273	Canada	PHASE2	50	Drug	Unknown	No
52	NCT04326959	Egypt	PHASE1|PHASE2	24	Combination	Unknown	No
53	NCT04391621	Uganda	PHASE2	30	Combination	Unknown	No
54	NCT04553159	Uganda	PHASE2	8	Biologic	Completed	No
55	NCT04582305	Netherlands	PHASE4	14	Drug	Unknown	No
56	NCT04593706	Brazil	N/A	40	Drug	Unknown	No
57	NCT04597060	Thailand	N/A	25	Combination	Unknown	No
58	NCT04710719	United States	N/A	7	Drug	Completed	No
59	NCT04722263	United States	N/A	15	Radiation	Recruiting	No
60	NCT04786210	United States	PHASE4	20	Combination	Completed	Yes
61	NCT04812626	Nepal	N/A	66	Drug	Completed	No
62	NCT04824612	Brazil	N/A	4	Combination	Completed	No
63	NCT04827875	United States	PHASE1	25	Drug	Completed	No
64	NCT04844840	United States	PHASE2	29	Drug	Completed	No
65	NCT04951869	Malaysia	PHASE2|PHASE3	90	Drug	Completed	No
66	NCT04988022	United States	PHASE4	44	Drug	Completed	No
67	NCT05072821	Taiwan	PHASE4	28	Drug	Completed	No
68	NCT05128383	United States	PHASE2	20	Drug	Completed	No
69	NCT05275699	China	PHASE1|PHASE2	25	Drug	Completed	No
70	NCT05330078	United States	EARLY_PHASE1	10	Drug	Completed	No
71	NCT05412745	Italy	N/A	64	Device	Completed	No
72	NCT05461157	United States	N/A	60	Device	Enrolling by invitation	No
73	NCT05488860	China	N/A	20	Combination	Unknown	No
74	NCT05887804	Indonesia	PHASE4	24	Combination	Completed	No
75	NCT05893108	Indonesia	PHASE1|PHASE2	46	Drug	Unknown	No
76	NCT05939817	Indonesia	PHASE4	24	Combination	Completed	No
77	NCT06138964	Singapore	PHASE2	50	Drug	Recruiting	No
78	NCT06230146	Egypt	PHASE1|PHASE2	45	Drug	Not yet recruiting	No
79	NCT06373458	United States	PHASE2	30	Drug	Recruiting	No
80	NCT06431360	China	N/A	80	Other	Recruiting	No
81	NCT06814288	Indonesia	PHASE2	20	Drug	Active not recruiting	No
82	NCT06909812	Mexico	PHASE3	40	Drug	Completed	No
83	NCT07014280	Egypt	PHASE4	28	Drug	Active not recruiting	No
84	NCT07263100	Thailand	N/A	20	Combination	Completed	No
